# Chlorpromazine–Polypyrrole Drug Delivery System Tailored for Neurological Application

**DOI:** 10.3390/molecules29071531

**Published:** 2024-03-29

**Authors:** Sara Krawczyk, Sylwia Golba, Cristina Neves, João Tedim

**Affiliations:** 1 Department of Science and Technology, Institute of Materials Engineering, Doctoral School, University of Silesia, Bankowa 14, 40-007 Katowice, Poland; 2 Department of Science and Technology, Institute of Materials Engineering, Bankowa 14, 40-007 Katowice, Poland; 3 Department of Materials and Ceramic Engineering, Centre for Research in Ceramics and Composite Materials (CICECO), University of Aveiro, Campus de Santiago, 3810-193 Aveiro, Portugal; cristina.neves@ua.pt (C.N.); joao.tedim@ua.pt (J.T.)

**Keywords:** drug delivery system, conducting polymers, polypyrrole, phenothiazine derivatives, heparin

## Abstract

Nowadays, drug delivery systems (DDSs) are gaining more and more attention. Conducting polymers (CPs) are efficiently used for DDS construction as such systems can be used in therapy. In this research, a well-known CP, polypyrrole (PPy), was synthesized in the presence of the polysaccharide heparin (HEP) and chlorpromazine (CPZ) using sodium dodecyl sulfate (SDS) as electrolyte on a steel substrate. The obtained results demonstrate the successful incorporation of CPZ and HEP into the polymer matrix, with the deposited films maintaining stable electrochemical parameters across multiple doping/dedoping cycles. Surface roughness, estimated via AFM analysis, revealed a correlation with layer thickness—decreasing for thinner layers and increasing for thicker ones. Moreover, SEM images revealed a change in the morphology of PPy films when PPy is electropolymerized in the presence of CPZ and HEP, while FTIR confirmed the presence of CPZ and HEP within PPy. Due to its lower molecular mass compared to HEP, CPZ was readily integrated into the thin polymer matrix during deposition, with diffusion being unimpeded, as opposed to films with greater thickness. Finally, the resulting system exhibited the ability to release CPZ, enabling a dosing range of 10 mg to 20 mg per day, effectively covering the therapeutic concentration range.

## 1. Introduction

In the elderly population, the incidence of neurodegenerative diseases such as Alzheimer’s (AD), Parkinson’s and Multiple Sclerosis is on the rise. These conditions lead to the damage of neurons in the human brain and neural system, resulting in mobility issues and cognitive impairment (dementia). Dementia is a particularly challenging symptom, affecting approximately 70% of AD patients [1]. Once neurons, which are the building blocks of the nervous system, are damaged or completely destroyed, they cannot be replaced. While they possess some regenerative capacity, extensive damage renders rebuilding impossible, leading to functional loss. As a result, there is an urgent need to alleviate the symptoms of neurodegenerative diseases [2]. The increase in the incidence of neurodegenerative diseases is associated with the accumulation of excessive amounts of specific proteins in the brain, namely beta-amyloid and tau. Beta-amyloid forms plaques that accumulate between neurons, disrupting cell function, while tau creates neurofibrillary tangles inside neurons, blocking the transmission of neuronal signals. The precise interplay between these proteins and the underlying causes of harmful layers formation, which leads to cell destruction, remain subjects of ongoing research [2]. Currently, there are only two types of medication aimed at treating symptoms. There are cholinesterase inhibitors that break down acetylcholine, a neurotransmitter crucial for intercellular signaling and memory retention. The other type of medication is NMDA receptor antagonists, such as memantine, which aim to block the effects of glutamate, a chemical known to cause nerve cell damage. Such pharmaceutical drugs can be used as the active components of drug delivery systems (DDSs).

One of the strategies currently in use for targeted delivery is the encapsulation of active compounds within a polymer matrix [3]. The polymer can act as responsive scaffold, with the embedded molecular drugs being released when an external trigger is applied. Conducting polymers (CPs) stand out amidst the polymers being considered for DDSs. CPs possess a range of advantageous features for DDSs, including high corrosion resistance, the capacity to change their conductivity in a controlled way and the ability to form thin films on various substrates [4,5]. So far, PPy has been amongst the most studied CPs with several works in the literature demonstrating the versatility of PPy for the development of DDSs for diverse applications, including anticancer drug delivery [6,7,8], the modulation of enzyme function and the detection of substances like lactate, glucose and glutamate in blood samples [9], as well as the controlled dosing of neuroleptic drugs like chloropromazine (CPZ) [10,11]. PPy has been studied in terms of usage in neural tissue applications. George at al. performed in vivo and in vitro studies to prove the ability of PPy to interact with neural tissue from the mammalian cerebral cortex. He compared the biocompatibility of a PPy implant with a stab wound. The results demonstrated the positive surface interaction between the PPy implant and cortical inference [12]. Also, in consideration of neurological application, the neurotoxic effect should be determined. The neurotoxic effect of a compound like CPZ was investigated in vitro using cell systems, for example, brain cell cultures. The biokinetics of CPZ should be taken into account. Broeders at al. conducted studies on aggregating rat brain cell cultures exposed to CPZ and diazepam (DZP) for 12 days. An analysis via HPLC-UV demonstrated that CPZ decreased over time in the medium, whereas the amount in the cells showed an increase. The accumulation of CPZ in the cells led to a higher intracellular concentration of this compound in the cells [13]. So far, heparin-conjugated fibers immobilized by the growth factors have been studied for neural cell growth. The results showed that the neural model cells (PC12 cells) were cultured well on the scaffold without inhibiting cell adhesion via heparin conjugation and exhibited cell proliferation. Such scaffolding showed promising nerve guidance conduits for promote peripheral nerve regeneration [14].

PPy is particularly attractive for neural applications as well due to its capacity of electrochemical fabrication into thin films and capabilities of loading drug substances during oxidation and potentially releasing them during electrochemical reduction [15]. Its redox behavior varies depending on the size of incorporated anions during electropolymerization. 

When small anions are placed on the film, they act only to compensate the charge of the oxidized sites in polymer. When larger anions are used, they are entrapped in the polymer matrix during reduction, and simultaneously, cationic species can then be inhaled. In the subsequent cycle, a release of mobile cations and solvent molecule motion occur. To compensate for the charge in oxidizing the PPy, heparin is commonly used as a negatively charged polysaccharide. 

Heparin (HEP) [16] is known for its anticoagulant properties that slow down the blood clotting process. When immobilized during electropolymerization on biomaterial, heparin enhances the hemocompatibility of the surface [17]. The synthesis of PPy with HEP has been shown to provide a sustainable substrate for endothelial cell growth, as demonstrated by Garner et al. [18,19]. 

In terms of CPZ delivery, some systems have been proposed in the literature, including intracellular drug delivery vehicles in the forms of cationic nanogels [20], self-nanoemulsifying drug delivery systems [21], and fast dissolving films (FDFs) for oro-buccal drugs [22]. 

The main goal of the present work was the development of a CPZ/HEP/PPy film on steel. The resulting film is expected to render multifunctional properties, namely, the controlled release of CPZ, an active pharmaceutical ingredient used in Alzheimer’s treatment, whereas HEP serves the dual purpose of binding the phenothiazine derivative CPZ to achieve a neutral charge state and inhibiting blood clotting. To the best of our knowledge, this innovative system has not yet been developed or studied, which highlights the originality of this research.

## 2. Results and Discussion

### 2.1. Synthesis of CPZ/HEP/PPy in Sodium Dodecyl Sulfate Solution

**Figure 1:**
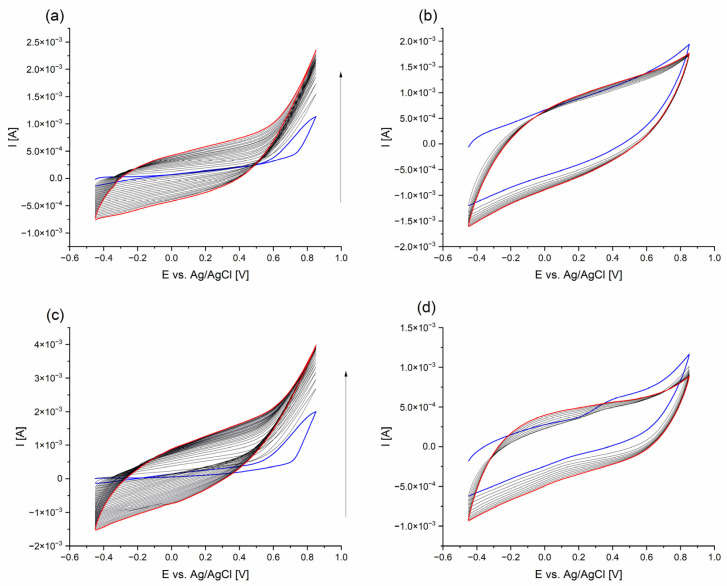
CPZ/HEP/PPy: (**a**) synthesis of f_1_; (**b**) cyclic voltammogram of f_1_; (**c**) synthesis of f_2_; (**d**) cyclic voltammogram of f_2_.

Cyclic voltammetry was used to synthesize PPy in the presence of CPZ and HEP, all within a sodium dodecyl sulfate solution. The role of SDS-based ions in the supporting electrolyte was twofold: to maintain the appropriate conductivity level and to extend the potential window of PPy synthesis, thereby enhancing its electrochemical activity [23,24]. The inclusion of saccharide-type HEP was driven by its anionic nature, which allowed it to serve as a polymeric counter ion, counterbalancing the positive charge generated during the growth of PPy chains [25]. At the same time, large HEP molecules became incorporated in the PPy matrix, interacting with CPZ molecules. The electropolymerization of CPZ/HEP/PPy 0.1:0.002:0.1 M in 0.1 M sodium dodecyl sulfate was induced as a result of cyclic scanning withing a prescribed potential range. Two maximum potentials were defined as follows: E_1_ = 0.85 V and E_2_ = 0.70 V. After reaching the maximum potential, reverse polarization occurred. This was evidenced by the formation of a black film layer covering the steel electrode. An exemplary cyclic voltammogram of CPZ/HEP/PPy is presented in Figure 1a, with the blue curve representing the first cycle, and the red curve, the last one. 

**Figure A1:**
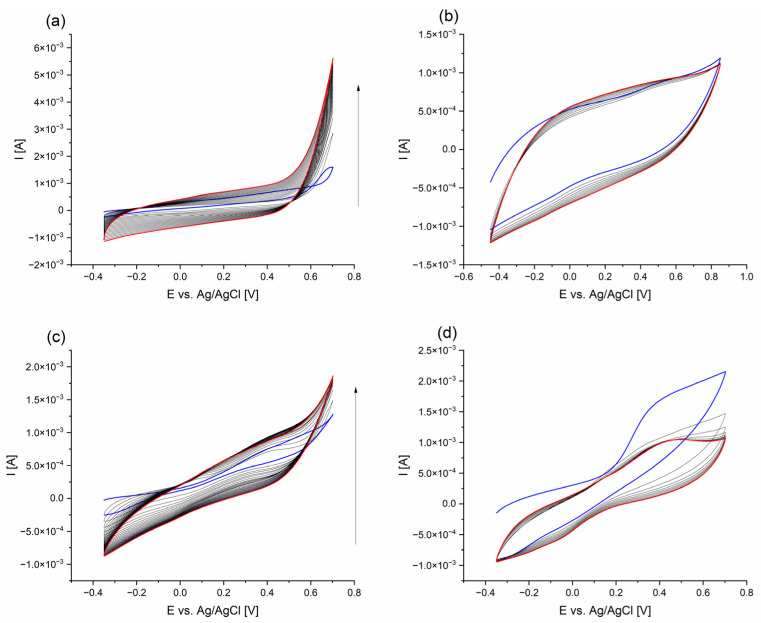
(**a**) Synthesis of f_3_; (**b**) cyclic voltammogram of f_3_; (**c**) synthesis of f_4_; (**d**) cyclic voltammogram of f_4_. Blue curve—first cycle and red curve—last cycle.

A cyclic voltammogram (CV) was used to characterize the deposition process for the CPZ/HEP/PPy system within the potential ranges of −0.45 to 0.85 V and −0.35 to 0.70 V, relative to the Ag/AgCl electrode (Figure A1). In the initial stage, the increase in potential triggered the oxidation of monomers, leading to a marked increase in the current intensity. This behavior is typical of pyrrole, as has been described in both organic [26,27] and aqueous [28,29] solutions. The value of monomer oxidation was found to be 0.7 V vs. SCE [30], corresponding to values reported by other authors, such as 0.65 V vs. Ag/AgCl (in SDS solution) [31] or 0.80 V vs. Ag/AgCl (in SDS solution) [32]. In accordance with the mechanism described in the literature, the oxidized monomer underwent a coupling reaction leading to the extension of the chain length, which adsorbed onto the surface of the steel electrode. In the following cycles, the process continued with an enhanced amount of deposited material having formed as a homogenous film. Furthermore, it demonstrated charge accumulation capabilities, as evidenced by the enhanced current intensity recorded in the subsequent cycles, indicating the formation of a conductive layer. The voltametric curves exhibited a repetitive shape with very broad oxidative (or reductive) branches, corresponding to the doping (and dedoping) of the forming PPy [33]. 

### 2.2. Electrochemical Properties of Polymers

Cyclic voltammograms (CVs) of the deposited films were recorded in a monomer-free electrolyte solution, namely in aqueous 0.1 M sodium dodecyl sulfate. The first cycle of CV curves (Figure 1) exhibited a slightly different pattern compared to the subsequent cycles, due to conditioning of the polymer. The subsequent curves overlapped, demonstrating the stability of the obtained layers in terms of charge accumulation and ion transfer capabilities.

The electrochemical behavior of the sample was evaluated by calculating a parameter called the stability index (ES) of the layer, which is based on the amount of charge exchanged during the doping cycle. This was calculated as the integral (area) under the current intensity curve, with reference to the time scale [34], in accordance with Equation (1):

(1) $$ES=\frac{Q^{+end}}{Q^{+2nd}}\cdot 100\%$$

where the variables are defined as follows:

*Q^+end^*—the charge of the last doping half cycle;

*Q^+2nd^*—the charge of the second doping half cycle.

The ES index served as a useful tool for comparing the doping ability of the synthesized films, and the results are presented in Table 1. 

The highest stability occurred in the synthesis performed with a higher number of cycles (30). Surprisingly, the calculated values exceeded 100%, which was presumably caused by two factors. The first factor is related to the thickness of the formed layers, which was larger with the increase in the number of polymerization cycles. A higher thickness may have led to the trapping of unpolymerized monomers or oligomer molecules in the free voids, which could then be released and subsequently polymerized in the subsequent CV cycles. This may result in an increase in the amount of material, thereby corresponding to an increase in the amount of accumulated charge. The second factor refers to the more effective movement of SDS anions during the doping process. As the CV was recorded in a solution of pure electrolyte, it may have facilitated the penetration of SDS into the polymer, thereby providing greater conductivity. It is known that using SDS as the base electrolyte solution may help PPy to retain its electrochemical activity within a given potential window. An increase in conductivity can also be associated with ion releasing during doping/dedoping processes [35], which is related to the ion mobility within a complex matrix. It is worth mentioning that, in the present work, the ES was larger than 100% whenever thick films (i.e., with 30 polymerization cycles) were prepared. 

It was stated by Halik et al. that porosity depends on the film thickness [36]. The thinnest film (0.04 µm) had a microporous structure (till 2 nm) for a PPy/DDS film. For the thicker films, a range from 0.2 to 1 µm with a mainly mesoporous structure and a 5 µm entirely mesoporous structure were considered. During electrochemical synthesis, a higher doping level (in the case of standard thick films) resulted in a more compact packing of PPy chains, and then, the density of the films increased, and there were smaller pores. In the present work, one of the goals was to entrap higher amounts of drug substance so that the standard and thick film were obtained. In the case of the standard film, the material was less porous, which resulted in a high stability (ES) around 100%. During the next cycles of polymerization, the structures of thick films changed to mesoporous trapping as well as monomers or oligomers, resulting in high stability values. Based on these statements [37], we can consider that changes in porosity occurred on the films. 

The theoretical dry coating thickness was estimated according to Equation (2) based on Faraday’s law:

(2) $$g=\frac{q_{polymer}\cdot M_{monomer}}{n\cdot F\cdot A_{w}\cdot \rho }$$

where the variables are defined as follows:

*g*—the thickness of the dry polymer film formed in the electropolymerization process, (cm); 

*q_polymer_*—the total charge of the polymerization process, expressed as the charge of the last polymerization cycle, (C);

*M_monomer_*—the molar mass of the monomer, (g·mol^−1^);

*n*—the number of electrons involved in the oxidation of one monomer unit, (2.3 for pyrrole [38]);

*F*—Faraday’s constant, (96.48 C·mol^−1^);

*A_w_*—the surface of the working electrode covered with a polymer film, (cm^2^);

*ρ*—approx. film density (1.48 g·cm^−3^) [39].

Given the wide range of polymer densities reported in the literature, ranging from 1.00 g·cm^−3^ [40] to 1.55 g·cm^−3^ [41], we opted for a value consistent with materials deposited under similar conditions, including the deposition technique. Consequently, we selected a density of 1.48 g·cm^−3^, as recommended in [42]. The thicknesses of the deposited films are shown in Table 1. The results confirm the impact of both the potential window and the extended duration of the deposition process. With an increase in the upper potential boundary (E_1_ vs. E_2_: 0.150 V difference), the film thickness increased 2.2 and 2.6 times for the 20 and 30 cycles, respectively. The thickness increase accompanying the extended time of the polymerization process (i.e., the increased number of polymerization cycles) was 1.7 times for the case when the applied potential was cycled up to more positive potentials (+0.85 V vs. Ag/AgCl), and only 1.5 times when the applied potential was cycled up to +0.70 V vs. Ag/AgCl. The obtained values are in line with the data obtained in other PPy studies, such as 1.18 μm for a salicylate-doped film on a copper surface [43], 5.8 μm for a a dexamethasone-doped film on a platinum disc electrode [44] and 2.7 μm for a toluene-4-sulfonic dopant, 5.4 μm for a camphor-10-sulfonic dopant or 14.5 μm for a hexafluorofosfate dopant [45]. PPy thicknesses of 5.0 ± 0.3 μm and 4.2 ± 0.2 μm were reported for PPy films doped with ClO_4_^−^ and SDS, respectively, and 7.3 μm prepared on ITO [46]. Film thicknesses of 3.5 and 3.3 μm for PPy-oxalic and PPy-DS, both deposited on iron substrates, have been reported [46,47]. 

Accompanying changes in the smoothness of the film influenced both by the deposition charge connected and experimental factors like the applied potential, cycle number, electrolyte solution and used substrate have been shown by Asplund [48].

### 2.3. Structural Analysis of PPy-Derived Films

**Figure 2:**
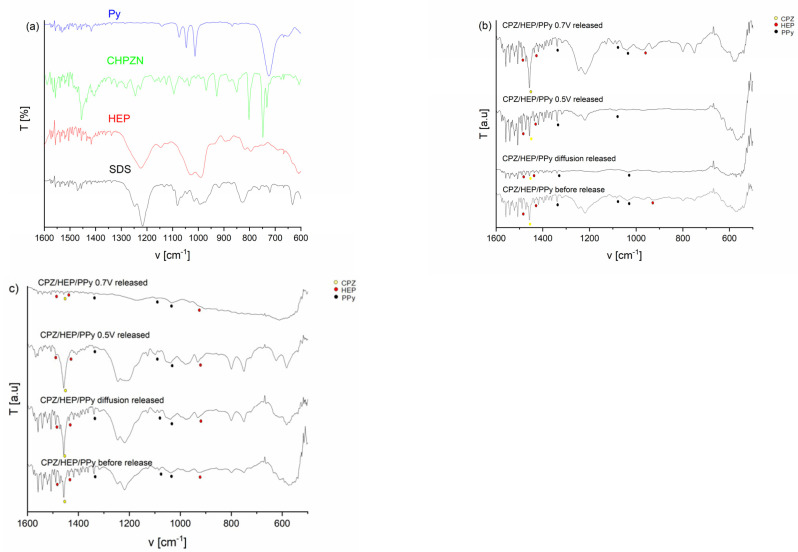
FTIR spectra: (**a**) reference samples. FTIR spectra of film before release, release with constant potentials of 0.5 V and 0.7 V and release without potential—diffusion: (**b**) f_1_; (**c**) f_3_, where yellow dot—CPZ, red dot—HEP and black dot—PPy.

**Figure A2:**
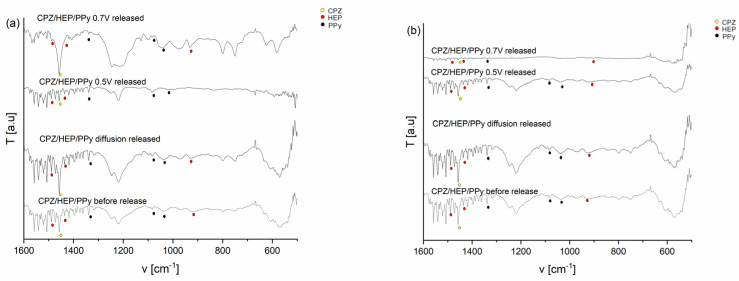
FTIR spectraof film before release, release with constant potentials of 0.5 V and 0.7 V and release without potential—diffusion: (**a**) f_2_; (**b**) f_4_; where yellow dot—CPZ, red dot—HEP and black dot—PPy.

FTIR spectra were acquired to obtain structural information of the films, both before and after the release of immobilized molecules. The spectra obtained prior to the release of CPZ/HEP are shown in Figure 2 (and Figure A2). Figure 2a shows reference samples in different colors: blue for pyrrole, green for CPZ, red for HEP and black for SDS.

For a qualitative analysis, the characteristic bands were identified to confirm the chemical structure of the material. In this range, Ppy-related bands were identified at 1313 cm^−1^, corresponding to the C-H in-plane deformation of PPy and N-H bending. Bands at 1074 cm^−1^, 1047 cm^−1^, 1012 cm^−1^ and 811 cm^−1^, attributed to the C-H wagging mode, along with a signal at 727 cm^−1^, were also detected. Bands at 1546 and 1452 cm^−1^ are associated with the fundamental vibration of the pyrrole ring, while the in-plane vibration of =C–H was observed at 1298 and 1039 cm^−1^. The vibration of the C–N bond corresponds to the band occurring at around 1196 cm^−1^. This analysis suggests a successful polymerization process resulting in PPy formation. Regarding the dopants, several bands correlating with the CPZ structure were observed. These include a band at 1456 cm^−1^ assigned to the skeleton vibration of the benzene groups characteristic of CPZ, and bands at 1070 cm^−1^ and 748 cm^−1^, corresponding to the stretching vibrations of C-S bonds [49]. Bands associated with HEP were detected at the following positions: 1487 cm^−1^, assigned to C=C stretching; 1430 cm^−1^, to the symmetric stretching of the carboxy group; and 932 cm^−1^, assigned to C-O-S stretching and C-O-C glycosidic bonds [50]. The analysis confirmed the incorporation of medical substances into the PPy matrix deposited on the steel substrate. For samples subjected to the desorption step, the recording of spectra was repeated. In this case, the characteristic bands belonging to both HEP and CPZ decreased in intensity, confirming the release of drugs from the PPy matrix.

**Figure 3:**
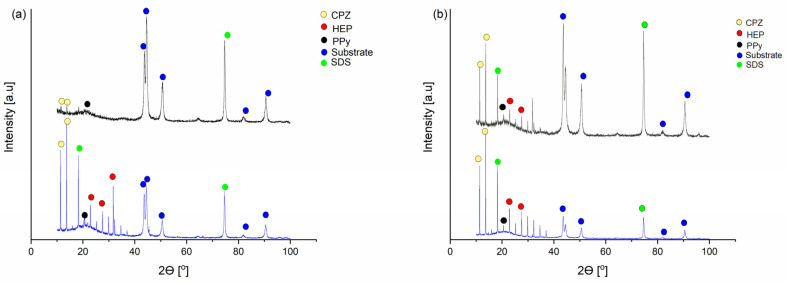
X-ray diffraction patterns of CPZ/HEP/PPy: (**a**) f_1_; (**b**) f_2_, before (blue) and after (black) the release of drug substances through application of constant potential of 0.7 V (yellow dot—CPZ, red dot—HEP, black dot—PPy).

X-ray diffractograms were recorded both before (blue line) and after the release (black curve) for samples f_1_ and f_2_, as shown in Figure 3a,b, respectively. The diffraction pattern of the PPy sample confirmed its predominantly amorphous nature, evidenced by a broad peak observed at 2θ = 24°. This peak was attributed to the scattering of PPy chains in the interplanar space [50]. The PPy sample was amorphous but had some structured areas. In using Scherrer’s equation (Equation (3)) [51], it is possible to estimate the average crystallite size:

(3) $$D=\frac{0.94\lambda }{\beta cos\theta }$$

where the variables are defined as follows:

*D*—crystallite size [nm]; 

*λ*—wavelength typical for Cu lamp 1.54 $\left[\dot{A}\right]$ [39];

*β*—full width at half maximum of angle of diffraction [radians];

*θ*—Bragg angle [52].

The calculated average crystallite size of the CPZ/HEP/PPy film was found to be 43 nm for f_1_ and 49 nm for f_2_. This is in line with the literature values, which report sizes ranging from 48 to 73 nm for PPy [50,51]. The range of variation in size observed in the literature is likely associated with the presence of SDS micelles in the solution [52].

A diffractogram of the CPZ/HEP/PPy film before the potential release of CPZ and HEP from f_1_ and after applying a constant potential 0.7 V is shown in Figure 3a. 

The intensity of CPZ peaks at 2θ = 11.9°, 15.9° [53], and the HEP 2θ = 26°, 28°, 33°, 35° [53] peaks decrease after release, confirming the expulsion of CPZ and HEP from the matrix. Broad peaks corresponding to PPy at 2θ = 20–25° [54,55] were visible, and their presence was consistent in both spectra. This broad peak is characteristic for PPy due to its amorphous structure and scattering of its chains. SDS peaks were observed at 2θ = 75° [56]. Substrate peaks corresponding to the substrate were seen at 2θ = 45° [57,58]. A decrease in intensity for the mentioned peaks of CPZ and HEP is visible in both diffractograms, but for the f_2_ film, it is less intense due to the impeded diffusion of drug substance molecules through the thicker polymer layer. 

### 2.4. Microstructural and Surface Properties of PPy-Derived Films 

**Figure 4:**
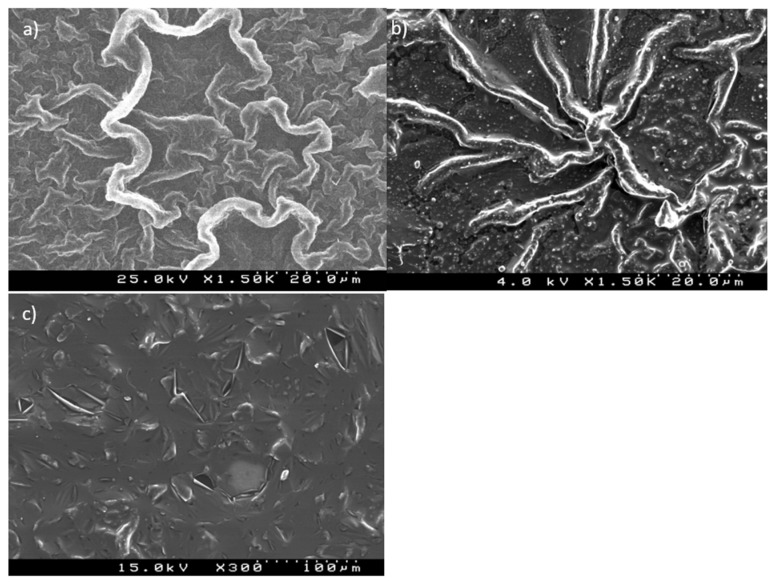
SEM images showing the microstructures of (**a**) HEP/PPy. SEM images of CPZ/HEP/PPy f_2_ (**b**) before the release of the drug substance and (**c**) after the release of the drug substance.

The microstructures of the HEP/PPy film and CPZ/HEP/PPy revealed through SEM are shown in Figure 4. 

The HEP/PPy (film synthesized in E = 0.85 V, 20c in SDS) (Figure 4a) microstructure demonstrated a PPy nanowire shape. The morphology and sizes of the PPy nanowires can be controlled through a ratio between PPy and HEP. The decrease in HEP concentration resulted in a reduction in nanowire length [59]. Prior to the release process, a characteristic “spider net” type of formation was observed for the CPZ/HEP/PPy film (Figure 4b). This structure was attributed to the aggregated skeleton of the SDS micelles, with particle sizes ranging from 46 to 100 nm for a single micelle [60,61]. SDS, being a surface-active compound, can form micelles above a certain critical concentration (CMC: 0.001 M) [50]. Its large agglomerates may adsorb onto the electrode surface. Generally, the negatively charged dodecyl sulfate DS molecular assemblies tend to repel each other, leading to the formation of extended structures resembling stars [62]. The molecular structure and organization of modified PPy doped with DS ions were described by A.V. Syugaev [63]. On iron substrates, an organized structure containing a large number of dodecyl sulfate anions was found. The agglomerates were coated with a PPy layer that was also spread over a flat space. The PPy was doped with spheres of interpenetrating heparin. The shapes of the adsorbed structures varied greatly depending on the local surface concentration near the electrode, which changed continuously during the polymerization process. Consequently, different heights of the given structures were observed, resulting in films with a non-uniform thickness and varying roughness [63].

The release of CPZ/HEP into the 0.9% NaCl solution resulted in dramatic changes to the microstructure. The image in Figure 4c reveals a much smoother surface with small crystal remnants of sodium chloride. Within the layer, some cracks are visible, likely produced by the inevitable ion migration as the material was immersed in saline solution. Additionally, it is known that transition from a non-conducting to a conducting state in CPs is associated with strains and volume changes [64,65], which may also contribute to the deformation. This process disrupts the uniformity of the layer and the previously observed “spider net” formation.

**Figure 5:**
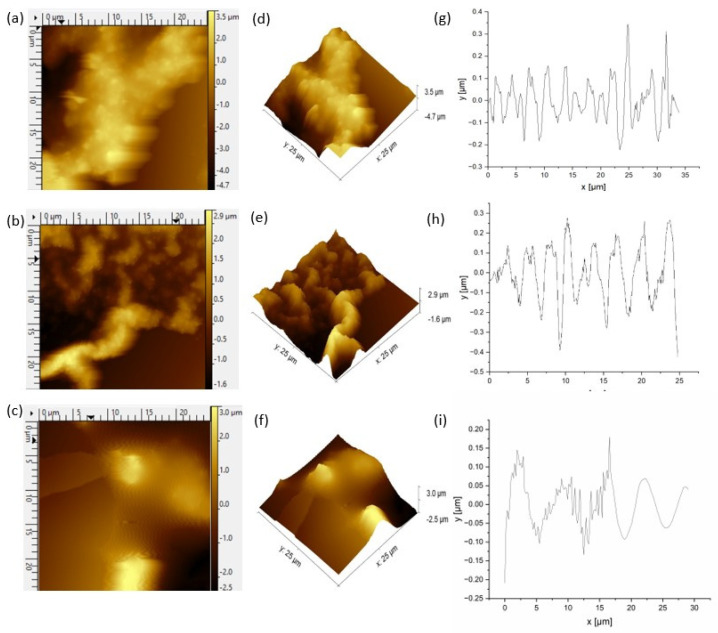
AFM images (25 µm × 25 µm) of (**a**) PPy film. AFM images of CPZ/HEP/PPy f_2_ (**b**) before and (**c**) after release of drugs substances. Topography of (**d**) PPy film. Topography of CPZ/HEP/PPy f_2_ (**e**) before and (**f**) after release of CPZ and HEP from PPy matrix. Roughness profiles of (**g**) CPZ/HEP/PPy film (**h**) before and (**i**) after release.

For the further analysis of the material structure, AFM images were recorded (Figure 5). 

These images not only provided insights into the topography but also enabled the calculation of the roughness of the obtained films. The AFM images correlated with the SEM microstructures, displaying the characteristic networks of the CPZ and PPy chains. The PPy coating covered the SDS based scaffold that was absorbed on the surface in the first stage of the process. Based on the AFM images, it can be presumed that the rate of DS^−^ adsorption was faster compared to the adsorption rate of the polymerization products, and allowed for the formation of such a composite. The roughness (R_a_) (Table 2) of the pure PPy synthesized in a separate experiment in SDS was 121 nm.

The R_a_ of film f_1_ before release was 171.12 nm, aligning with typical values found for PPy-based drug-doped layers: 57.58 nm for nanostructured PPy [penicillin/streptomycin] on Ti, 100.04 nm for PPy [dexamethasone] on Ti [66], 100–200 nm for hybrid PPy doped with dexamethasone [67] and 160 nm for DS-doped PPy [68]. HEP-doped PPy also exhibited roughness values of 118 and 58.2 nm, depending on the content of polysaccharide (concentration 0.20 vs. 0.40 g/L), revealing a dendritic porous morphology [69]. According to A.V. Syugaev, thin, multilayered plates of PPy grown in 2D are vertically oriented. These studies indicated that the rings of PPy are arranged in-plane with the substrate, and the long chain molecules are oriented normal to it, contributing to the obtained morphology [70].

The release procedure resulted in a decrease in roughness to a value of 137 nm, representing a 20% reduction in roughness. This change was associated with the expulsion of previously entrapped molecules, including both the drug and the polysaccharide.

A different trend was observed in the thicker film (30 cycles) (images not included) where the roughness increased after the release stage from an initial value of 70.52 nm to 153.8 nm (an increase of 218%). This behavior can be attributed to the movement impediments encountered by the entrapped molecules in the dense, thick polymer matrix. The application of an external constant potential led to changes in volume and state of order and pushed the dopants to be expelled, which in turn increased the observed roughness. The mobility of these dopants was restricted due to steric and diffusional constraints. Therefore, in the studied timeframe, the release was limited by these internal factors.

### 2.5. Release Studies 

**Figure 6:**
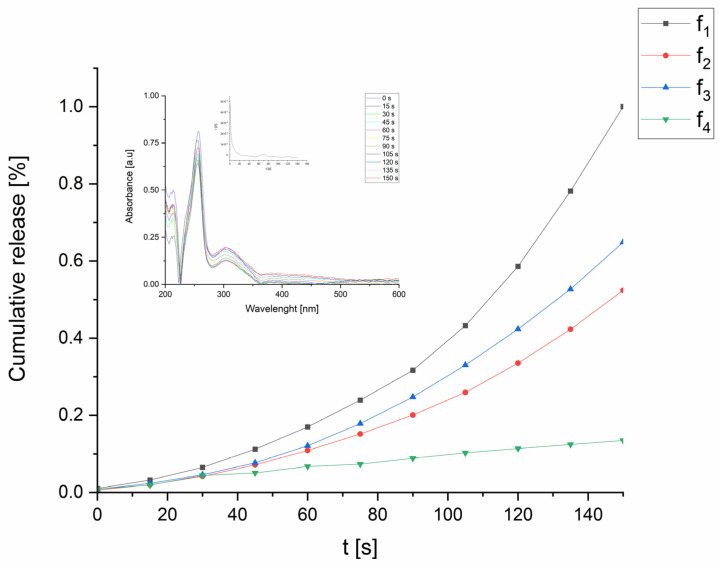
Cumulative release of CPZ/HEP/PPy released at +0.7 V vs. Ag/AgCl. The color codes for the spectra are as follows: black for f_1_; red for f_2_; blue for f_3_; and green for f_4_. Inserted are the UV-Vis spectra of the chlorpromazine release from f_2_ and its chronoamperogram.

In the final stage, UV-Vis spectroscopy was coupled with chronoamperometry to stimulate and trace the release of the drug from the film previously obtained via CV. During the procedure, the film was conditioned for 120 s to stabilize the polymer film. Then, a constant potential of +0.7 V (vs. Ag/AgCl) was applied for 150 s. UV-Vis spectra of the test solution were recorded, both at the initial time and after 150 s. A cumulative release plot, depicting the release of CPZ in the physiological solution versus time, is shown in Figure 6. 

UV-Vis spectra of the CPZ released in the physiological solution showed an increase in absorbance for the characteristic bands of CPZ at λ = 253 and HEP λ = 213 nm, respectively, during the release process. For this system, the encapsulation efficiency (EE) was calculated using Equation (4) [44]:

(4) $$EE=\left[\frac{concentration\;of\;drug\;in\;polymer\;film}{concentration\;drug\;in\;polymer\;solution}\right]\cdot 100\%$$

The entrapment efficiency was calculated for all films. Svirskis et al. released progesterone from PPy films and showed an EE maximum value of 0.062% in sodium nitrate solution [71]. The highest EE (Table 3), exceeding 2%, was observed for film f_2_. A direct comparison with the literature data is handicapped due to the different methods used, concentration of PPy and compositions. The data shown in the introduction section indicate that a similar entrapment efficiency was obtained.

Conversely, the lowest EE was recorded for film f_4_, where the applied potential likely generated short oligomers that were insufficient for binding CPZ effectively to the matrix. The results show that thick films were able to entrap more of the drug substance compared to thinner ones. 

Moreover, the active substance release efficiency was obtained by calculating the ratio of the concentration of the substance in the solution after release (c_3_) to the concentration of the active substance initially introduced into the matrix during the synthesis (c_1_). Importantly, c_1_ was calculated from the difference between the initial concentration of the active substance in the solution (c_0_) and its remaining concentration after synthesis (c_2_). The calculated parameters for each released dopant molecule are presented in Table 3.

The highest ratio (RE) of CPZ release was observed for the film f_3_, while the lowest release ratio was noted for f_2_. CPZ, having a smaller molecular mass compared to HEP, showed enhanced incorporation into the matrix. 

HEP was released more efficiently when polymerization was conducted up to higher oxidizing potentials (+0.85 V vs. Ag/AgCl, E_1_), as it is shown in Table 3. This observation is related to the length of the PPy chains, where shorter chains are not capable of effectively attaching to the high molecular mass of HEP. It is connected with the applied synthesis condition, which also influences the film structure that was discussed in Section 2.3 [44,70,71]. The presence of the large heparin ion also affected the release process. In terms of the heparin RE, a higher potential of synthesis led to a higher release efficiency, which was greater for f_1_ than for f_2_ (77 vs. 9.5%). It manifested the presence of longer chains of PPy that entangled more strongly with HEP, and hence decreased the HEP RE. The difference in the thickness also supports this view, as for f_2_, it was equal to 13.5 µm, and f_1_ was 7.8 µm. It can also manifest the presence of additional porosity in the system. For the samples synthesized at the lower potentials of f_3_ and f_4_, the lengths of the forming chains were shorter and did not allow for a similarly strong HEP immobilization, so the HEP release was more efficient. 

According to the literature, to achieve a therapeutic effect while using a drug, patients typically need to take daily doses of CPZ ranging from 10 to 75 mg. However, daily dosages can even go up to 300 mg and, in some cases, exceed 800 mg [72]. We estimate, tentatively, that our proposed systems could ensure the dosing of 10 mg and 20 mg per day (calculated based on one 1 cm^2^ of deposited sample), covering part of the range of the concentrations necessary for therapeutic effects.

## 3. Materials and Methods

### 3.1. Materials

The synthesis was performed in an aqueous medium containing the following ingredients: pyrrole (98%, Sigma Aldrich, Schnelldorf, Germany), chlorpromazine CPZ (>98% TLC, Sigma Aldrich, Schnelldorf, Germany) (structure in Table 4), heparin sodium salt from porcine intestinal mucosa HEP (>180 USP units/mg, Sigma Aldrich, Schnelldorf, Germany) and sodium dodecyl sulphate (>99%, Sigma Aldrich, Schnelldorf, Germany). Prior to synthesis, pyrrole was pre-distilled for purification, resulting in a transparent, pale, yellow liquid, which was then stored in a freezer. The electrolyte solution was prepared by dissolving SDS in distilled water to achieve a concentration of 0.1 M, surpassing the critical micelle concentration of 0.008 M [50]. The monomer solution was prepared at a concentration of 0.2 M, with the molar ratio of the components CPZ:HEP:PPy set at 1:0.002:1. To avoid inhomogeneities, the synthetic solution was freshly prepared just before the experiment. The stainless steel substrate (316L) was used due to its relevance for biomedical applications (composition: Cr 16%, Ni 10%, Mo 2%, C 0.08%, P 0.045%, S 0.03%, Si 0.75%, N 0.1%). Stainless steel was used as a working electrode (WE) prepared by polishing with sandpaper (ranging from grit 300 to 600, Klingspor, Bielsko Biała, Poland) and washed with distilled water to remove any powder impurities. Subsequently, the substrate was immediately immersed in a 60% ethanol solution for 24 h to prevent the formation of an oxide layer. All measurements were conducted at room temperature.

### 3.2. Polymerization of PPy-Derived Films via Cyclic Voltammetry and Chronoamperometry Procedures Used to Stimulate the Release of Entrapped CPZ and HEP

The measurements were carried out in an electrochemical cell containing a reference Ag/AgCl electrode, a counter electrode (platinum spiral) and a 316L steel plate as a working electrode. Cyclic voltammetry (CV) was employed for the synthesis and characterization of the deposited polymeric films. CV analysis was performed using an Autolab PGSTAT12 potentiostat, and the results were analyzed with Autolab software (version 4.9). During the synthesis, two maximum potential values were applied, E_1_ = 0.85 V and E_2_ = 0.70 V, to detect their influence on the deposition process as well as on the drug immobilization efficiency. All potentials referred to the Ag/AgCl reference electrode. To stimulate the release of the drug substance, chronoamperometry was used, with a potential set at either 0.5 or 0.7 V, held for 150 s.

### 3.3. Characterization of CPZ/HEP/PPy Films

Fourier transformed infrared (FTIR) spectroscopy was performed using a Shimadzu IR Prestige 21 Fourier Spectrometer to identify the functional groups and verify the material composition. Prior to the analysis, the samples were rinsed with distilled water to remove any residual oligomers. Subsequently, they were gently heated under inert gas conditions (up to 60 °C for a duration of 12 h). The central region of each sample was chosen as the test area. Spectra were recorded in the 1600–600 cm^−1^ range with a resolution of 2 cm^−1^ with adjustments made for moisture and carbon dioxide interferences in the optical path.

X-ray diffraction patterns of the coated samples were collected at room temperature using a Panalytical X’Pert PRO diffractometer: Bragg–Brentano geometry, Ni-filtered Cu Kα radiation, a PIXcel1D detector and step of 0.02°. The exposure corresponded to the angular range of 2θ between 10 and 100° with a scan step size equal to 0.026 s for Cu anode. 

Scanning electron microscopy (SEM) was carried out using a Hitachi S4100 model equipped with energy dispersive X-ray spectroscopy (EDS) with an acceleration voltage of 15 keV. For these analyses, samples were firmly fixed onto a steel sample holder using adhesive carbon tape (SEM Conductive Double sided Carbon Tape, Extra Pure, width: 5 mm, micro-shop). Additionally, the surface of the PPy film was coated with an additional layer of carbon powder using an EMITECH K950X carbon coater to guarantee a conducting surface for the analysis. 

Atomic force microcopy was used to characterize the surfaces of the films by capturing high-resolution nanoscale images. The analysis was carried out in non-contact mode with a scan rate of 0.4 Hz on a Multimode atomic force microscope (Nanoscope IV from Veeco, Plainview, NY, USA). The AFM tips used were non-coated NCH-50 cantilevers (Non-contact/Tapping™ mode—High resonance frequency) from a Pointprobe—silicon (SPM Sensor). These tips had a thickness of 4 µm and a resonance frequency of 320 kHz. The widest scanning area of the samples were 50 × 50 μm. An analysis of the images was performed using Gwyddion 2.58.

The UV-Vis measurements were carried out using a quartz cuvette (Bionovo, Legnica, Poland) in a Biowave II UV-vis spectrometer (WPA, Biochron, Legnica, Poland). For the release stage, the coated samples were fully immersed in a 0.9% NaCl solution and placed along the side wall of the cuvette to allow the diffusion of drug molecules into the bulk of the solution. The absorption band of CTZ was located at 260 nm [75], while the HEP absorption band was located at 213 nm [76,77]. The spectra were recorded in real time, with readings taken every 15 s, while a constant potential was applied using the AUTOLAB PGSTAT12 potentiostat. The chosen potentials values were based on the respective potentials observed in the CV doping/dedoping cycles.

## 4. Conclusions

In the process of electropolymerization, CPZ/HEP/PPY drug delivery systems were successfully developed on 316L steel substrates. These systems demonstrated high electrochemical stability. The spectral analysis with FTIR confirmed the successful incorporation of drug substances during synthesis. SEM microstructural analysis revealed a characteristic “spider net” pattern in the PPy, which changed with the release of CPZ and HEP. AFM measurements showed that the roughness of the sample decreased for 20 cycles but increased for 30 cycles after release, resulting in rougher surfaces that may favor heterogeneous nucleation. HEP was more efficiently incorporated during the 20-cycle synthesis, due to its high molecular mass. It was released more efficiently at the E_1_ potential than at E_2_, as shorter chains formed at E_1_ were less capable of entrapping and enclosing HEP, facilitating a better release. This resulted in a higher active substance release efficiency (RE) at E_1_ compared to E_2_. CPZ, with its lower molecular mass, incorporated more easily into the polymer matrix during the 20-cycle synthesis, where the diffusion process was not blocked by the film thickness, unlike in the 30-cycle synthesis. This led to a lower concentration of CPZ with E_1_ and 30 cycles. According to the literature, the therapeutic effects of CPZ are achieved with daily doses ranging from 10, 25, 50 to 75 mg [62]. Our proposed systems demonstrated the capacity for the dosing of CPZ at 10 mg and 20 mg per day. As this covers a portion of the necessary concentration range for therapeutic effects, these results are promising. They represent a significant step toward developing systems capable of delivering a wider range of dosages, potentially aligning with the full spectrum recommended for therapeutic efficiency.

## Figures and Tables

**Table 1:** Calculated electrochemical stability index (ES) and thickness (g) parameters of synthesized CPZ/HEP/PPy films.

Synthesis Parameters	Name	ES [%]	g [µm]
E_1_ = 0.85 V, cycles 20	f_1_	92.50	7.80
E_1_ = 0.85 V, cycles 30	f_2_	105.64	13.50
E_2_ = 0.70 V, cycles 20	f_3_	94.080	3.52
E_2_ = 0.70 V, cycles 30	f_4_	110.52	5.20

**Table 2:** Roughness (R_a_) of CPZ/HEP/PPy films before and after the release of drugs substances.

Film	R_a_ before Release [nm]	R_a_ after Release [nm]
f_1_	171.12	137.00
f_2_	70.52	153.80
f_3_	158.23	146.030
f_4_	50.21	89.24

**Table 3:** Active substance release efficiency (RE) (explanations in the footer) and encapsulation efficiency (EE) of CPZ in PPy films. The table also includes the encapsulation efficiency of CPZ in PPy films with standard deviation, calculated based on UV-Vis spectra data.

Synthesis Parameters	Film	CPZ RE [%]	HEP RE [%]	EE CPZ [%]	m CPZ Released [mg/cm^2^]
E_1_ = 0.85 V, c. 20	f_1_	75 ± 0.18	77 ± 0.13	0.64 ± 0.020	15.26 ± 0.12
E_1_ = 0.85 V, c. 30	f_2_	56 ± 0.090	9.5 ± 0.020	2.01 ± 0.050	20.96 ± 0.20
E_2_ = 0.70 V, c. 20	f_3_	82 ± 0.23	11 ± 0.080	0.96 ± 0.070	17.79 ± 0.18
E_2_ = 0.70 V, c. 30	f_4_	68 ± 0.12	17 ± 0.090	0.37 ± 0.010	11.30 ± 0.080

The RE was calculated as the ratio of c_3_ to c_1_, where c_3_ represents the concentration of the substance in the solution after release, and c_1_ is the concentration of the active substance introduced into the matrix during synthesis. The value of c_1_ was derived from the difference between the initial concentration of the active substance in the solution (c_0_) and its concentration after synthesis (c_2_). Values were recalculated to percentages.

**Table 4:** Molecular and structural formulas of SDS and CPZ.

Name	Molecular Formula	Molecular Formula
SDS	C_12_H_25_NaO_4_S	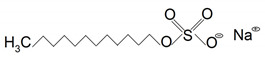 [73]
CPZ	C_17_H_19_CIN_2_S	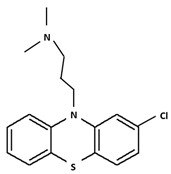 [74]

## Data Availability

Data are contained within the article.
